# Oral treatment with 10% potassium iodide solution for refractory cutaneous-disseminated sporotrichosis in an immunocompetent adult: Case report

**DOI:** 10.3389/fmicb.2022.994197

**Published:** 2022-10-28

**Authors:** Kaiwen Zhuang, Yaling Dai, Yike Zhou, Yujing Ke, Xin Ran, Yuping Ran

**Affiliations:** ^1^Department of Dermatology, West China Hospital, Sichuan University, Chengdu, China; ^2^Laboratory of Dermatology, Clinical Institute of Inflammation and Immunology, Frontiers Science Center for Disease-Related Molecular Network, West China Hospital, Sichuan University, Chengdu, China; ^3^Department of Lab Medicine, West China Hospital, Sichuan University, Chengdu, China; ^4^West China Hospital, West China School Medicine, Sichuan University, Chengdu, China

**Keywords:** cutaneous disseminated sporotrichosis, *Sporothrix globosa*, potassium iodide, itraconazole, voriconazole (VCZ)

## Abstract

Sporotrichosis has multiple clinical manifestations, and its cutaneous-disseminated form is uncommon and, in most cases, related to immunosuppressive conditions. We report the case of a 47-year-old male patient who presented with multiple cutaneous nodules and ulcers on the left upper limb and the right thigh, with no other comorbidities. Until the diagnosis was confirmed, the patient was initially given empiric antifungal treatment with itraconazole, which showed unsatisfactory results at a local hospital. Then, he was treated with voriconazole, which led to the slow improvement of his skin lesions. At one point during the voriconazole treatment course, the patient briefly self-discontinued voriconazole for economic reasons, and the lesions recurred and worsened. The patient was finally diagnosed with cutaneous-disseminated sporotrichosis based on the isolation and identification of *Sporothrix globosa*. Susceptibility testing revealed that the isolate was resistant to itraconazole, fluconazole, voriconazole, terbinafine, and amphotericin. Considering the patient's poor financial condition, potassium iodide was administered. After 1-month of therapy with potassium iodide, he reported rapid improvement of his skin lesions. The patient continued potassium iodide treatment for another 5 months until the full resolution of lesions was achieved.

## Introduction

Sporotrichosis is a sub-acute to chronic subcutaneous mycosis caused by the ubiquitous, thermodimorphic fungus, *Sporothrix* complex (Valeriano et al., 2020). It occurs worldwide, predominantly in tropical and subtropical countries, such as Mexico, Central America, South America, and Africa (Barros et al., 2011). According to the immune status of the host, the load and location of the inoculation, and the thermal tolerance of the strain, sporotrichosis presents a series of clinical manifestations, which are clinically categorized into fixed cutaneous, lymphocutaneous, cutaneous-disseminated, and extracutaneous forms (Bonifaz and Tirado-Sánchez, 2017). Lymphocutaneous sporotrichosis is the most common form, while the cutaneous-disseminated form is uncommon and is mostly related to immunosuppressed individuals (Severo et al., 1999; Bonifaz and Vazquez-Gonzalez, 2010). Herein, a rare case of cutaneous-disseminated sporotrichosis in an immunocompetent man is presented.

## Case presentation

A 47-year-old male patient presented to our hospital with multiple cutaneous nodules and ulcers on the left upper limb and the right thigh. He worked as a farmer in a rural region and had no pathological history. The lesions initially appeared 4 years ago on his middle finger of the left hand, where trauma occurred when he cut yak meat, and then, it gradually spread to the rest of his left upper limb and the right thigh. Over the past 4 years, he was hospitalized two times at a local hospital for presumed cutaneous invasive fungal infection and non-tuberculous *Mycobacterium* infection and had received empiric treatment with itraconazole, rifampicin, and levofloxacin for nearly 2 years with no obvious improvement. Then, the patient presented to the infection department of the West China Hospital and was hospitalized.

Blood investigations revealed an elevated erythrocyte sedimentation rate of 24 mm/h, an absolute CD3 lymphocyte count of 937 cell/μl, and an absolute CD8 lymphocyte count of 237 cell/μl. Absolute CD4 lymphocyte count, white blood cell count, and neutrophil percentage were normal. TB interferon-gamma release assay (TB-IGRA) was positive. Other blood investigations, including liver function, renal function, HIV, and viral hepatitis screening, were normal. Ultrasonography of the abdomen was also normal. Chest computed tomography (CT) revealed several sub-centimeter pulmonary nodules without lymphadenopathy. The culture of skin species for bacteria revealed *Staphylococcus epidermidis*, while that for fungi and mycobacteria was negative. Silver methenamine stain from biopsy tissue revealed several suspicious fungal spores. Detection of the skin tissue by the next-generation sequencing (NGS) was negative for bacteria, viruses, fungi, and mycobacteria.

**Figure 1:**
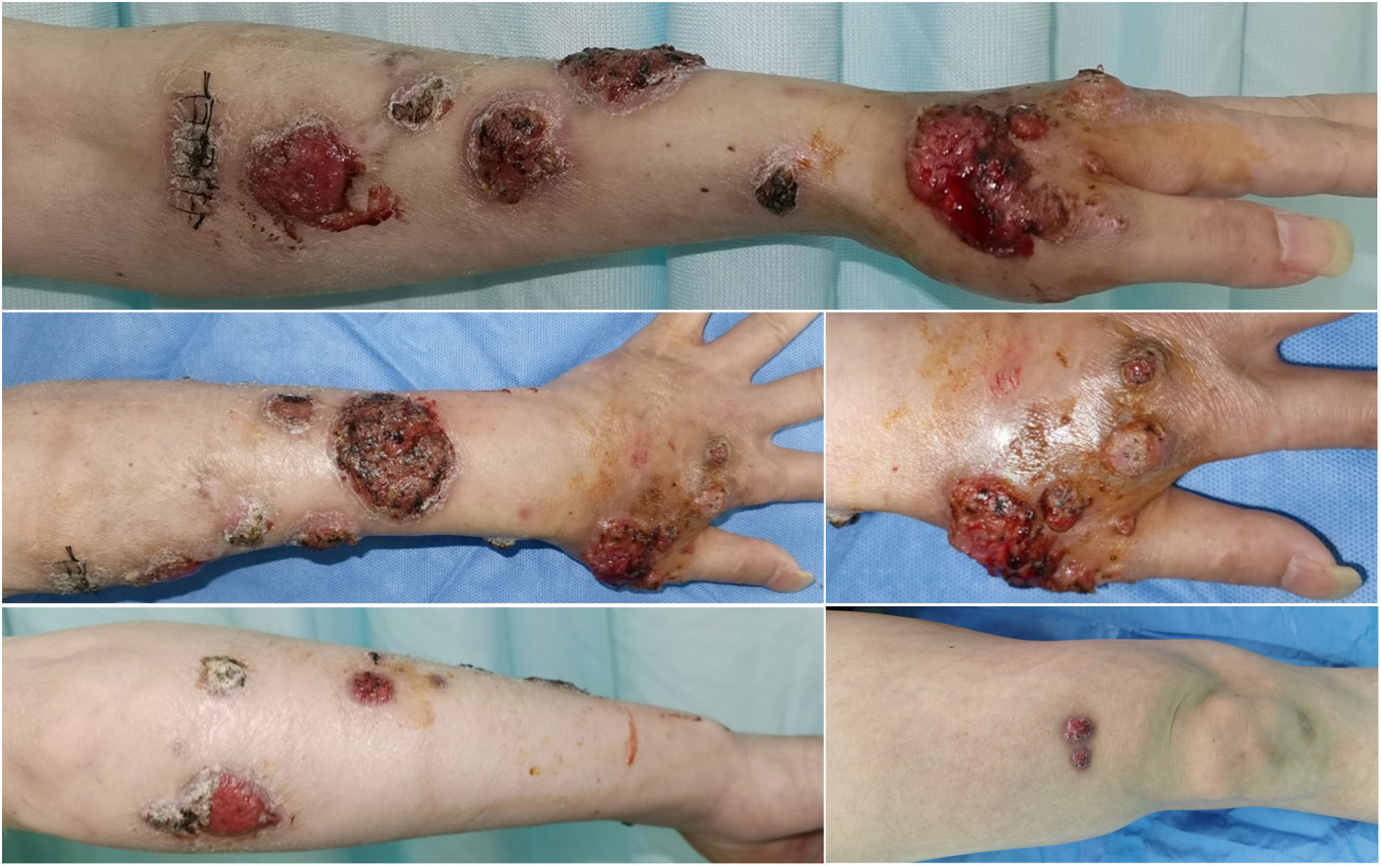
Multiple cutaneous lesions caused by *Sporothrix globosa*. A 47-year-old man with multiple verrucous and ulcerated nodules with overlying necrotic eschar on left upper limb, scattered crusted papules and plaques on right thigh.

**Figure 2:**
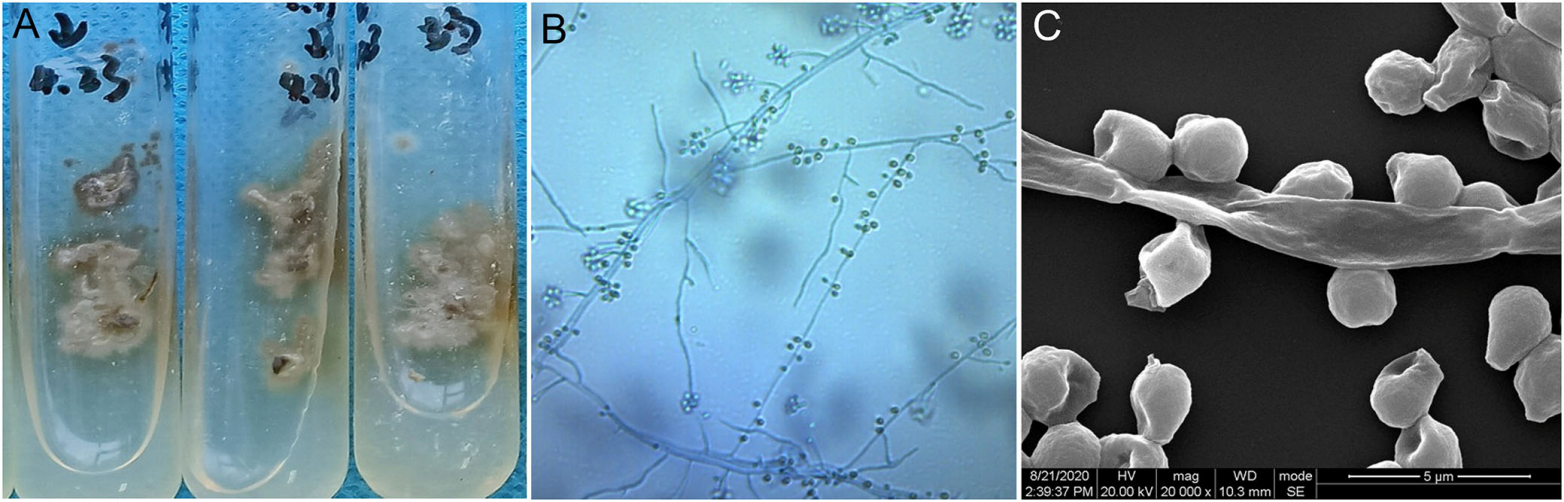
**(A)** Culture of *Sporothrix globosa* (Sabouraud dextrose agar, 28°C) grew grayish white colonies. **(B,C)** Slide culture and scanning electron microscope observations revealed thin hyphae and denticle microconidia like “daisy flowers”.

Before empiric antifungal treatment, the patient was referred to the dermatology clinic for screening for cutaneous fungal infection. Physical examination revealed large scattered verrucous and ulcerated nodules with overlying necrotic eschar on the left upper limb. Further examination of the trunk and extremities revealed scattered crusted papules and plaques on the right thigh (Figure 1). After skin tissue culture for fungi was performed in the dermatology clinic, the patient was discharged and started on empiric oral voriconazole 400 mg daily. The fungal culture on sabouraud dextrose agar (SDA) at 28°C for 10 days grew into grayish-white colonies (Figure 2A). Microscopic examination with slide culture and scanning electron microscope observations of the colony indicated the morphology of *Sporothrix* spp. (Figures 2B,C). The strain was identified as *Sporothrix globosa* by calmodulin gene (CAL) sequence analysis.

**Figure 3:**
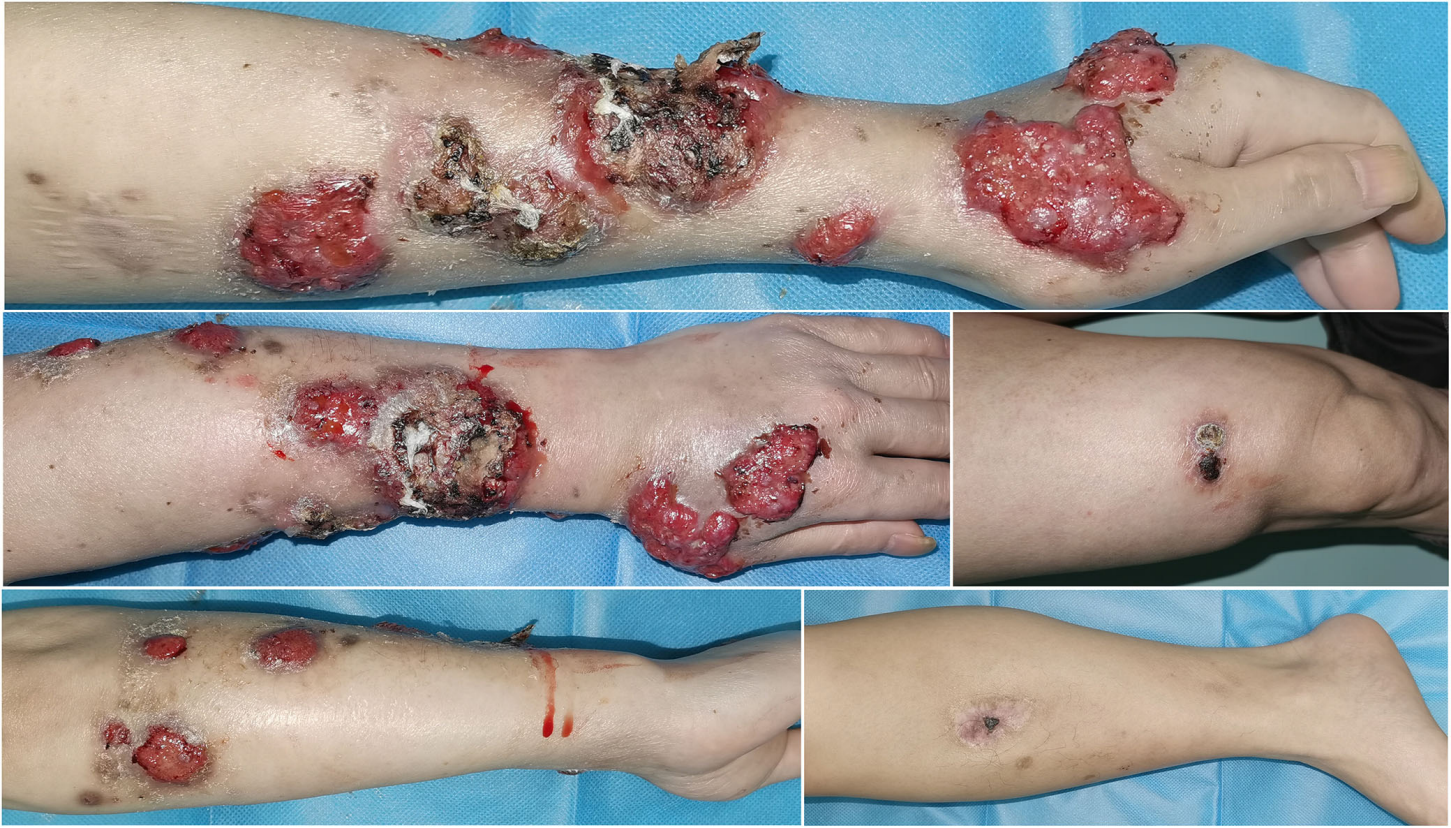
After self-discontinuation of voriconazole, the existing lesions (verrucous and ulcerated nodules on the left upper limb, and papules and plaques on the right thigh) recurred and worsened, extending to the right leg with crusted plaque.

**Figure 4:**
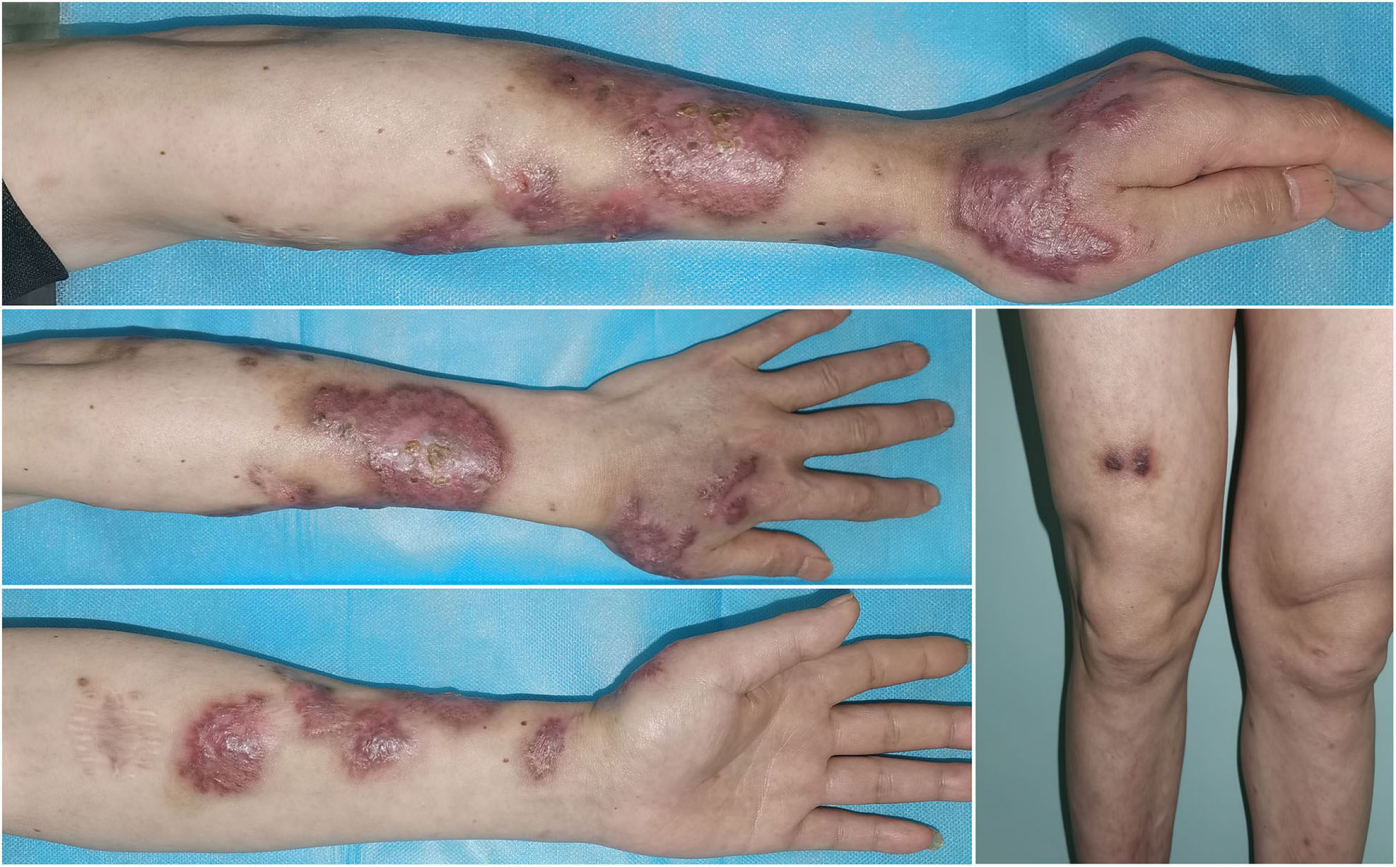
After 6-month therapy of potassium iodide, the patient achieved complete remission with only scarring remaining.

Based on the aforementioned evidence, a diagnosis of cutaneous-disseminated sporotrichosis was finally established, but the patient had not shown up for follow-up but continued antifungal therapy with voriconazole at a local hospital. During a telephone follow-up, the patient reported a slow improvement after voriconazole treatment. After 8 months of antifungal treatment, the patient self-discontinued voriconazole due to financial constraints, and the lesions recurred and worsened (Figure 3). He visited our dermatology clinic again for further treatment. An antifungal susceptibility test was performed by using the E-test (BIO KONT, China), which revealed that the isolate in this case was resistant to itraconazole, fluconazole, voriconazole, terbinafine, and amphotericin. Meanwhile, considering the patient's poor financial condition, a 10% solution of potassium iodide was administered. After 1-month of therapy with potassium iodide, there was a rapid improvement in his skin lesions. He continued potassium iodide treatment for another 5 months until there was a complete resolution of lesions (Figure 4). There was no recurrence at the 6-month follow-up.

## Discussion

Cutaneous-disseminated sporotrichosis (CDS) is characterized by multiple skin lesions at non-contiguous sites without extracutaneous involvement. Lesions of the fixed and lymphocutaneous forms may coexist in the same patient (Barros et al., 2011). The entity identified in this patient is a rare form of sporotrichosis, which only accounts for <1.75–8% of cases of *Sporothrix* infections (Song et al., 2013; Garcia et al., 2021). In China, the incidence of CDS is even lower. The cutaneous-disseminated form represented only 0.34% (14/4,969) of all sporotrichosis cases in a large-scale clinical epidemiological investigation of sporotrichosis reported from China (Lv et al., 2022).

CDS in most cases affffects immunodefificient individuals, frequently related to patients with HIV, hematologic cancer, diabetes mellitus, steroid treatment, chronic alcoholism, malnutrition, those who are pregnant and had undergone transplantation (Bonifaz and Tirado-Sánchez, 2017). There are few reports of immunocompetent individuals with disseminated lesions (Almeida-Paes et al., 2014), as in this patient. Dissemination in immunocompetent hosts has been linked to cat scratches, which cause multi-site repeat inoculations (Barros et al., 2011; Bonifaz and Tirado-Sánchez, 2017).

A literature search was performed in the PubMed database using the item “disseminated cutaneous sporotrichosis” for cases reported from January 2002 to June 2022 (Table 1). Of the 52 published cases of CDS found from the review, 35% were women and 65% were men, with an average age of 45.7 years (range 5–76 years). Among them, 28 patients were from Brazil, eight from the United States, five from Mexico, four from Malaysia, and two from China. HIV, diabetes, alcoholism, and a history of cat contact are the common predisposing factors. In the review, 22 published cases of CDS occur in hosts without obvious immunocompromised conditions.

**Table 1:** Summary of reports on CDS in immunosuppressed and immunocompetent patients.

**Time**	**Country**	**Age/sex**	**Risk factor**	**Site of primary lesion**	**Clinical manifestations**	**Diagnostic method**	**Pathogen**	**Treatment**	**Outcome**	**References**
2017	Brazil	45/male	Cat scratch	Upper limbs	Ulcerated nodules	Culture	*Sporothrix globosa*	ITZ	Improved	Queiroz-Telles et al., 2022
2022	Brazil	52/male	Minor occupational injury	Hands, right forearm, eyes, feet, legs, buttocks	Ulcerated nodules	Culture, PCR	*Sporothrix schenckii*	ITZ	Cure	Queiroz-Telles et al., 2022
2022	Malaysia	50/male	Gardening and contact with cats	Fac, trunk, extremities	Multiple nodules	Culture, histopathology	*Sporothrix schenckii*	AMB, ITZ	Unknown	Seow et al., 2022
2022	Japan	76/male	IgG4-related disease, prednisolone therapy	Forearms, upper back	Irregularly-shaped dark red plaques, ulcers	Culture, PCR, histopathology	*Sporothrix globosa*	ITZ, TRB	Cure	Nomoto et al., 2022
2022	PRC	55/female	Tuberculous peritonits	Knee, arms, left leg, hands, knees, left wrist	Erythematous and broken lesions	Culture, PCR, histopathology	*Sporothrix globosa*	ITZ	Improved	Shi et al., 2022
2021	Mexico	21/male	ND	Chest, abdominal wall, arms, forearms	Multiple ulcerated nodules	Culture, histopathology	*Sporothrix schenckii*	KI	Improved	Martínez-Herrera et al., 2021
2021	USA	37/female	Gardening, heart surgery	Posterior aspect of right elbow	Multiple nodules, arthralgias	Culture, PCR, histopathology	*Sporothrix schenckii*	ITZ	ND	Garcia et al., 2021
2020	Brazil	41/female	Kidney-pancreas transplantation, diabetes	Cutaneous, oral and nasal mucosa	ND	Culture, PCR, histopathology	*Sporothrix brasiliensis*	AMB, ITZ, TRB	Cure	Fichman et al., 2021
2020	Brazil	43/male	Renal-transplant-recipient	Nose, upper lips, scalp, dorsum, oral and nasal mucosa	Ulcerated and crusted nodules, molluscum-like papales	Culture, PCR, histopathology	*Sporothrix brasiliensis*	AMB-L + ITZ	Death	Fichman et al., 2021
2020	Brazil	61/female	Cat scratch and diabetes mellitus	Upper limbs, trunk, face	Verrucous and ulcerated plaques	Culture, histopathology	*Sporothrix brasiliensis*	AMB, ITZ	Cure	Valeriano et al., 2020
2020	Brazil	26/female	Cat bites	Arms, hands, fingers	Multiple nodules	Culture, histopathology	*Sporothrix brasiliensis*	ITZ	Cure	Valeriano et al., 2020
2020	Brazil	64/male	Type 1 diabetes	Hands, arm, elbow	Erythematous nodules and ulcers	Culture, histopathology	*Sporothrix brasiliensis*	ITZ, local heat	Cure	Valeriano et al., 2020
2020	Brazil	46/female	Cat scratch	Back, left arm, face	Ulcerated nodules, lymphangitis	Culture, histopathology	*Sporothrix brasiliensis*	ITZ	Cure	Valeriano et al., 2020
2020	Mexico	45/male	Bucket striking	Distal third of the right leg, both lower legs	Ulcers	Culture, PCR, histopathology	*Sporothrix schenckii*	ITZ	Cure	Alvarez-Rivero et al., 2020
2020	Brazil	38/female	HIV infection, cats contact	Hands, back, face	Erythematous papules, pustules, ulcers, crusts	Culture, PCR, histopathology	*Sporothrix brasiliensis*	AMB, ITZ	Cure	Poester et al., 2020
2020	Brazil	56/male	Alcoholism	Left wrist, face, scalp, left arm, skin	Erythematous and ulcerated nodules	Culture, histopathology	*Sporothrix* species	AMB, ITZ	Improved	Valente et al., 2020
2019	USA	62/male	Play golf	Left lateral thigh, left posterior thigh	Erythematous ulcers	Culture, PCR, histopathology	*Sporothrix schenckii*	AMB, PSZ,ITZ, TRB	Cure	White et al., 2019
2019	USA	35/female	Cats contact, alcoholism, diabetes	Right forearm, legs, contralateral arm, abdomen	Erythematous nodules, ulcerations	Culture	*Sporothrix schenckii*	AMB, PSZ,ITZ	Cure	Saeed et al., 2019
2018	Peru	42/male	ND	Face, limps, arms, legs	Erythematous and verrucous papules, plaques	Culture, histopathology	*Sporothrix schenckii*	KI	Improved	Rueda et al., 2018
2018	Brazil	13/female	ND	Throughout the body	Ulcerative lesions	Culture, PCR, histopathology	*Sporothrix brasiliensis*	ITZ	Improved	Fernandes et al., 2018
2018	Japan	47/male	Ulcerative colitis	Right lower leg, left pretibial area	Red nodules, ulcer	Culture, histopathology	*Sporothrix globosa*	KI, local heat	Cure	Takazawa et al., 2018
2017	USA	57/female	Asthma, an arthropod bite	Left elbow, left upper arm	Ulcers, fevers, chills, fatigue	Culture, histopathology	*Sporothrix schenckii*	ITZ	Improved	Charles et al., 2017
2017	Brazil	39/female	Scratched by cat	Abdomen skin	Multiple sites ulcers	Culture, PCR, histopathology	*Sporothrix brasiliensis*	AMB, ITZ	Cure	Lima et al., 2017
2017	Brazil	47/male	Alcoholism	Left leg, limbs, trunk, abdomen, scalp	Cutaneous softened nodules, subcutaneous masses	Skin biopsy, mycological examination	*Sporothrix*	KI	Cure	Benvegnú et al., 2017
2017	USA	65/female	Chronic lymphocytic leukemia	Lip, left nares, left cheek, left arm, leg, upperback	Vegetative plaque, crusted papules, plaques	Culture, histopathology	*Sporothrix schenckii*	PSZ,ITZ	Cure	He et al., 2017
2017	Brazil	34/male	Alcoholism, HIV, cat contacts	Torso, face, chest, extremities	Annular brownish papules, reddish shallow ulcers	Culture, histopathology	*Sporothrix*	AMB, ITZ	Improved	de Oliveira-Esteves et al., 2017
2017	Brazil	35/male	HIV positive	Cutaneous, osteoarticular, oral, nasal mucosa, left eye	Diffuse, ulcerated, crusty nodules	Culture, PCR	*Sporothrix brasiliensis*	AMB,ITZ	Cure	Biancardi et al., 2017
2017	Brazil	25/male	HIV positive, direct trauma	Cutaneous, osteoarticular, pulmonary, bone marrow, lymph nodal, eyes	Diffuse, ulcerated, crusty nodules	Culture, PCR	*Sporothrix brasiliensis*	AMB,ITZ	Cure	Biancardi et al., 2017
2017	Brazil	43/male	HIV positive, cat contacts	Cutaneous, osteoarticular, eyes	Diffuse, ulcerated, crusty nodules	Culture, PCR	*Sporothrix brasiliensis*	AMB,TRB	Cure	Biancardi et al., 2017
2016	Brazil	59/female	Cat scratch	Face, left cervical, upper limbs	Ulcerated nodules, lymphadenopathy	Culture	*Sporothrix*	ITZ	Cure	Medeiros et al., 2016
2016	Zambia	27/female	HIV positive	Nose, upper limbs, trunk	Skin rash, papules, ulcerated plaques	Histopathology	*Sporothrix schenckii*	ITZ	Improved	Patel et al., 2016
2015	Brazil	5/male	ND	Face, gluteal region, upper and lower limbs	Nodular erythematous skin lesions	Culture, histopathology	Sporothrix schenckii.	AMB, ITZ	Cure	Ribeiro et al., 2015
2015	Mexico	68/male	Alcoholism	Face, thorax, abdomen, limbs, head	NODULES, plaques	Culture, histopathology	*Sporothrix schenckii*	ITZ	Cure	Cotino Sánchez et al., 2015
2013	USA	41/male	HIV, alcoholism, cutaneous trauma	Left hand, other body sites	Nodules	Culture, histopathology	*Sporothrix schenckii*	ITZ	ND	Chang et al., 2013
2013	Brazil	39/female	ND	Left foot, lower limb, upper arm, groin, abdomen, back	Papules, nodules, ulcers	Culture, serology	*Sporothrix schenckii*	AMB, ITZ	Improved	Eustace et al., 2013
2013	USA	53/male	Hepatitis C, alcoholism	Chest, head, trunk, legs, arms	Erythematous, ulcers	Culture, histopathology	*Sporothrix schenckii*	ITZ	Improved	Sharon et al., 2013
2012	Malaysia	61/male	ND	Whole body	Ulcers	Culture, histopathology	*Sporothrix schenckii*	AMB, ITZ, TRB	Death	Tang et al., 2012
2012	Malaysia	71/female	ND	Face, upper limbs, lower limbs	Ulcerated nodules and plaques	culture, histopathology	*Sporothrix schenckii*	AMB, ITZ	Improved	Tang et al., 2012
2012	Brazil	59/male	HIV	Cutaneous, conjunctival mucosa	Papules, nodules, conjunctivitis	Culture	*Sporothrix schenckii*	ITZ	Cure	Freitas et al., 2012
2012	Brazil	27/male	HIV	Cutaneous, meningoencephalitis	Plaque, papale	Culture	*Sporothrix schenckii*	ITZ,AMB	Cure	Freitas et al., 2012
2012	Brazil	46/female	HIV	Cutaneous, osteoarticular, oral, nasal mucosa	Plaque, papale	Biopsy	*Sporothrix schenckii*	ITZ,AMB	Cure	Freitas et al., 2012
2012	Brazil	26/male	HIV	Cutaneous, meningoencephalitis	Large cystic masses	Culture, biopsy	*Sporothrix schenckii*	ITZ,AMB	Death	Freitas et al., 2012
2012	Brazil	47/male	HIV	Cutaneous, osteoarticular	Plaque, papale, nodule	Culture	*Sporothrix schenckii*	ITC,AMB	Cure	Freitas et al., 2012
2012	Brazil	44/male	HIV	Cutaneous (wide spread), nasal mucosa	Plaque, papale, nodule	Culture	*Sporothrix schenckii*	AMB	Cure	Freitas et al., 2012
2011	PRC	36/male	Pleurisy	Left leg, trunk, limbs	Nodules, abscesses, ulcers	Culture	*Sporothrix schenckii*	ND	ND	Zhang et al., 2011
2011	Brazil	52/male	Alcoholic hepatopathy	Left thigh, the rest of his body	Papules, nodules, ulcers, molluscum-like lesions	Culture, histopathology	*Sporothrix schenckii*	AMB, ITZ	Death	Schechtman et al., 2011
2011	Malaysia	70/female	Gardening, cat contacts	Face, upper and lower limbs	Ulcerated nodules	Culture, histopathology	*Sporothrix schenckii*	AMB, ITZ	Cure	Yap, 2011
2011	Mexico	36/male	ND	Dorsum and anterior abdomen	Papular lesion, ulcers	Culture, PCR, histopathology	*Sporothrix schenckii*	AMB,ITZ;KI	Cure	Romero-Cabello et al., 2011
2006	Mexico	74/male	ND	Anterior right wrist	Skin lymph nodes	Culture, histopathology	*Sporothrix schenckii*	ITZ	Unknown	Campos-Macías et al., 2006
2006	USA	40/male	Blackberry picking	Trunk, upper extremities, left arm	Ulcers, lesions	Culture, histopathology	*Sporothrix schenckii*	ITZ	Improved (almost healed)	Yang et al., 2006
2003	Brazil	24/male	HIV, alcoholism	Left leg, face, thorax, arms	Lesions	Culture, histopathology	*Sporothrix schenckii*	ITZ	Improved	Carvalho et al., 2002
2002	USA	72/male	Diabetes	Left hand, abdomen, left elbow	Ulcer, nodules	Culture	*Sporothrix schenckii*	ITZ	Cure	Stalkup et al., 2002

AMB, amphotericin B; ITZ, itraconazole; TRB, terbinafine; PSZ, posaconazole; KI, potassium iodide; ND, no data available.

Diagnosis of cutaneous-disseminated sporotrichosis is challenging due to its diverse clinical manifestations. The condition can affect any part of the body surface, presenting cutaneous features that include numerous ulcerated nodules and verrucous plaques (Saeed et al., 2019). This polymorphic presentation is distinct from the classic “sporotrichoid” appearance of the most common lymphocutaneous form of sporotrichosis. CDS can extend to mucous membranes, bones, joints, various organs, and systems and rapidly progress to fungemia (Bonifaz and Tirado-Sánchez, 2017).

Diagnosis of CDS is often delayed or misdiagnosed because its diverse clinical symptoms are easily confused with other conditions such as PG, Sweet's syndrome, tuberculosis, sarcoidosis, and other mycotic or parasitic infections, including cutaneous leishmaniasis (Saeed et al., 2019). Culture from tissue fragments, exudative lesions, scales, sputum, and blood remains a gold standard for diagnosis. Culture using sabouraud dextrose agar, incubated at 25–30°C, is a standard technique applied in most cases, but it is time-consuming (Barros et al., 2011). The histopathologic features of granulomatous inflammation with cigar-shaped organisms and asteroid bodies are supportive but have low sensitivity (Barros et al., 2011).

Itraconazole and amphotericin B are the most useful therapies for patients with CDS, as in the current review (Saeed et al., 2019; Valeriano et al., 2020). In refractory cases, different combination therapies can be considered. Potassium iodide, an inexpensive and fairly safe preparation, has been found to be consistently effective against *Sporothrix*. Potassium iodide and itraconazole in combination with thermotherapy are preferred therapeutic options in cutaneous-disseminated cases of sporotrichosis (Valeriano et al., 2020). The treatment with potassium iodide alone or combined with hyperthermia has also been reported in CDS, as in our case and the four published cases described in the review (Benvegnú et al., 2017; Rueda et al., 2018; Takazawa et al., 2018; Martínez-Herrera et al., 2021).

In summary, CDS is an uncommon clinical form of infection caused by *Sporothrix*, and it is even rarer in immunocompetent hosts. Due to the increased incidence of the condition, it is significant to maintain a high degree of suspicion in the presence of lesions similar to that reported here. A fungal culture is crucial to confirm the diagnosis of CDS. Although itraconazole and amphotericin B have been recommended for CDS, potassium iodide is a safe and effective alternative.

## Data availability statement

The original contributions presented in the study are included in the article/supplementary material, further inquiries can be directed to the corresponding author.

## Ethics statement

The studies involving human participants were reviewed and approved by Biomedical Research Ethics Committee of West China Hospital of Sichuan University. The patients/participants provided their written informed consent to participate in this study.

## Author contributions

YR and KZ contributed to conception and design of the study. YZ and YK organized the database. XR and YD performed the statistical analysis. KZ wrote the first draft of the manuscript and sections of the manuscript. All authors contributed to manuscript revision, read, and approved the submitted version.

## Funding

This research was supported by the Natural Science Foundation of China (No. 81803150) and the HX-Academician project (HXYS19003) of West China Hospital, Sichuan University.

## Conflict of interest

The authors declare that the research was conducted in the absence of any commercial or financial relationships that could be construed as a potential conflict of interest.

## Publisher's note

All claims expressed in this article are solely those of the authors and do not necessarily represent those of their affiliated organizations, or those of the publisher, the editors and the reviewers. Any product that may be evaluated in this article, or claim that may be made by its manufacturer, is not guaranteed or endorsed by the publisher.
